# Illuminate the hidden: in vivo mapping of microscale pH in the mycosphere using a novel whole-cell biosensor

**DOI:** 10.1038/s43705-021-00075-3

**Published:** 2021-12-11

**Authors:** Bi-Jing Xiong, Christian Dusny, Lin Wang, Jens Appel, Kristin Lindstaedt, Dietmar Schlosser, Hauke Harms, Lukas Y. Wick

**Affiliations:** 1 Department of Environmental Microbiology, Helmholtz Centre for Environmental Research-UFZ, Permoserstraβe 15, 04318 Leipzig, Germany; 2 Department of Solar Materials, Helmholtz Centre for Environmental Research-UFZ, Permoserstraβe 15, 04318 Leipzig, Germany; 3 Key Laboratory of Urban Environment and Health, Institute of Urban Environment, Chinese Academy of Sciences, Xiamen, 361021 China; 4 University of Chinese Academy of Sciences, 100049 Beijing, China; 5 Department of Biology, Christian-Albrechts-Universität zu Kiel, Am Botanischen Garten 5, 24118 Kiel, Germany

**Keywords:** Fungi, Biological techniques

## Abstract

The pH of an environment is both a driver and the result of diversity and functioning of microbial habitats such as the area affected by fungal hyphae (mycosphere). Here we used a novel pH-sensitive bioreporter, *Synechocystis* sp. PCC6803_peripHlu, and ratiometric fluorescence microscopy, to spatially and temporally resolve the mycosphere pH at the micrometre scale. Hyphae of the basidiomycete *Coprionopsis cinerea* were allowed to overgrow immobilised and homogeneously embedded pH bioreporters in an agarose microcosm. Signals of >700 individual cells in an area of 0.4 × 0.8 mm were observed over time and used to create highly resolved (3 × 3 µm) pH maps using geostatistical approaches. *C. cinerea* changed the pH of the agarose from 6.9 to ca. 5.0 after 48 h with hyphal tips modifying pH in their vicinity up to 1.8 mm. pH mapping revealed distinct microscale spatial variability and temporally stable gradients between pH 4.4 and 5.8 over distances of ≈20 µm. This is the first in vivo mapping of a mycosphere pH landscape at the microscale. It underpins the previously hypothesised establishment of pH gradients serving to create spatially distinct mycosphere reaction zones.

## Introduction

Despite the high motility of protons, pH gradients over even short distances (µm-mm) may be both driver and the result of localised microbial activity in spatially structured microhabitats [1–5] such as the mycosphere, biofilms [6], biocrusts [7], soil-biochar surfaces [8] or the rhizosphere [9]. Although increasing numbers of studies report on organisms shaping their specific microbiome by adjusting the local pH [10, 11], it remains unclear at which scale single organisms may influence their surrounding pH.

Over the last few years, approaches to analyse pH at high spatial resolution have been published. For instance, techniques involving nanoparticles [12, 13], needle-type microelectrodes [14, 15], or planar optodes [7] have been developed for the in vivo analysis of pH. While nanoparticle-based sensors allow for horizontal and vertical pH profiles at the nanoscale, they are not yet commercially available, and professional expertise is needed for their synthesis. Commercial microelectrodes typically have needle tips of ~8 µm diameter and allow for pH analysis at approximately the 10 µm scale. They are, though, highly invasive, mainly when applied for mapping the pH distribution (ca. 100 insertions, for instance, are needed to cover a surface area of 1 mm^2^) or time-lapse monitoring of a given area. Being poorly invasive, planar optodes, by contrast, can be used for spatiotemporal analysis of, for example the pH development in the rhizosphere [9]. However, having a typical sensing resolution of >150 µm, they are not suitable to resolve pH heterogeneity at the scale often relevant for cellular functioning [16, 17]. For the first time, we here complement existing pH analysis approaches by genetically constructing a robust whole-cell bacterial pH bioreporter (*Synechocystis* sp. PCC6803_peripHlu) that allows for spatial and temporal in vivo analysis of environmental pH at the single-cell scale (~3 µm). *Synechocystis* sp. PCC6803 was chosen for transformation due to its ability to grow photoheterotrophically with glucose as optional carbon and energy source [18]. This characteristic allows the broad application of the transformant in both carbon source rich and poor environments. Using the Tat pathway [19], the ratiometric pH-sensitive GFP variant reporter protein pHluorin2 [20, 21] was translocated to the periplasm of *Synechocystis* sp. PCC6803, which is thought to have the same pH as the cell surrounding even at rapid environmental pH shifts [22]. Ratiometric pHluorin2 displays a bimodal excitation at 395 nm and 475 nm with maximum emission at 510 nm [21]. Upon acidification, emission at 510 nm after excitation at 395 nm (I_510–395_) decreases, yet increases after excitation at 475 nm (I_510–475_). The 510 nm emission intensity ratio from two excitations (R_I510–475/I510–395_, abbreviated as R_I475/I395_) thus increases in response to decreasing environmental pH. pHluorin2 is a well-known pH sensor that has been introduced to cells and tissues for sensing intracellular pH [23–25], yet to our knowledge has never been applied to report extracellular pH. Many filamentous fungi lower their ambient pH through the excretion of organic acids for various purposes [26, 27]. Mycosphere processes [28] involved in the degradation of organic matter, lignin and lignocellulose, the bioavailability and transport of nutrients [29], mineral weathering or soil structure changes are often pH-dependent. We hence tested the *Synechocystis* sp. PCC6803_peripHlu biosensor to dynamically quantify hyphal modifications of the environmental pH at the microscale. Hyphae of the filamentous agaric litter decay basidiomycete *Coprionopsis cinerea* [30], were allowed to overgrow homogeneously distributed bioreporter cells incubated on microscopy-compatible agarose pads to reveal spatial and temporal acidification profiles of the mycosphere caused by the basidiomycete. *C. cinerea* was chosen as a representative of fungal – often basidiomycete – litter degraders largely contributing to plant litter decomposition in upper soil layers [31]. Lignocellulose-decaying basidiomycetes are in particular known to excrete and employ pH-lowering and metal (Mn, Fe)-chelating organic acids such as oxalate, malate or malonate in order to meet the specific pH and reactant requirements of their respective extracellular plant cell wall-degrading machineries [32, 33]. Combining time-resolved quantitative ratiometric signals of >700 spatially distributed bioreporters with geostatistical approaches, we created pH maps to visualise spatial and temporal dynamics of pH distribution in the mycosphere of *C. cinerea* at a resolution of 3 × 3 µm. The area around mycelia forms an important and widespread habitat of high microbial activity [34] and interactions [35, 36]. Therein, microscale pH gradients have been proposed as important drivers for biogeochemical processes, such as the solubilisation and mobilisation of nutrients and metals [7, 34, 37] or the enzymatic decomposition of organic compounds [38]. Most experimental studies, however, only refer to the bulk pH and neglect a detailed analysis of pH at the microscale. It, however, seems unlikely that bulk soil pH (i.e. averaged over grams or millimetres to centimetres) may reflect the habitat pH of individual microbial cells. Instead, pH perceived by single-cell bioreporters at micrometre scale allows a mechanistic understanding of microbial habitat properties and functioning.

## Methods and materials

### Construction of *Synechocystis* transformants expressing periplasmic pHluorin2

The reporter protein pHluorin2 is a ratiometric pH-sensitive GFP variant [21]. The construct for periplasmic expression of pHluorin2 was obtained by cloning the TAT-specific targeting signal of TorA from *E. coli* at the N-terminus of pHluorin2, similar to Spence et al. [19]. To this end, the primer pairs G-5-trc + 3-trc-rbs, N-Luorin2 + C-luorin2, and 5-Apra + G-3-Apra (Supplementary Table S1) were used to amplify PCR fragments containing the *trc*-promoter, the pHluorin2 gene and the cassette for apramycin resistance, respectively. All three fragments were inserted by Gibson cloning [39] into the vector pBS_slr0168 that was cut by XhoI and PstI in a single step. The resulting construct was cut with NdeI, and a PCR product generated with the primers N-TorA3 and C-TorA3 (Supplementary Table S1) was inserted by Gibson cloning. Subsequently, the obtained constructs (pBS-TorpHluorin) were verified by sequencing and transformed into wild type cells of *Synechocystis* sp. PCC6803 according to standard protocols [40]. The vector directs homologous recombination to the putative silent site as described by Kunert et al. [41]. The extracellular pH sensitivity of *Synechocystis* transformants was calibrated microscopically; i.e. R_I475/I395_ of >300 bioreporter cells was quantified during exposure to both unbuffered and buffered microcosms (cf. Supplementary Tables S2 and S3 for composition of media) adjusted to pH values between 4.0 and 8.2.

In order to assess the effect of the growth phase of *Synechocystis* bioreporter cells for environmental pH reporting, a growth experiment of 16 days (cf. SI for details) was performed to analyse the stability of cellular R_I475/I395_ at pH 7 over time.

### Microorganism, medium and growth conditions

*Synechocystis* sp. PCC6803_peripHlu was grown at 30 °C on a rotary shaker at 150 rpm with an illuminating light intensity of 50 µmol photons m^−2^ s^−1^, in flasks containing 20 mL of YBG 11 medium (pH 7.2, Supplementary Table S2, supplemented with 100 ppm apramycin, buffered with 50 mM HEPES). At the mid-exponential growth phase (OD_750_ = 3.1), 0.15 mL of the cell culture was harvested and centrifuged for 5 min at 7000 g. The supernatant was discarded. The cell pellet was then resuspended in one mL of YBG 11 medium (pH 7, adjusted by 2 N H_2_SO_4_; cf. Supplementary Table S2 for composition) to obtain a suspension of OD_750_ = 0.45. The suspension was then further applied as inoculum to the bioreporter agarose pad in the microcosms (cf. below). *C. cinerea* was used as filamentous fungus [30]. It was cultivated at 25 °C for 3 days on yeast-malt extract-glucose medium. Using a hollow cylinder (Ø 8 mm), an agarose piece was cut from the peripheral growth zone and used as inoculum to the microcosms (cf. below).

### Microscale pH sensing: microcosm setup, description and experimental procedure

**Fig. 1:**
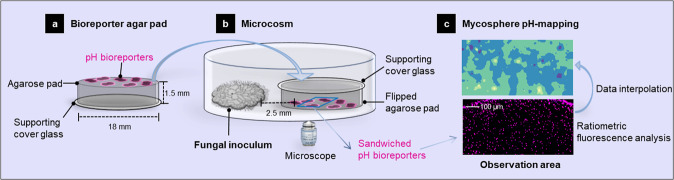
Schematic view of the microcosm used for the cultivation and microscopic quantification of pH by whole-cell bioreporters. **a** Agar pad inoculated with a monolayer of pH-sensitive bioreporters. The pad was put upside down to place the bioreporter monolayer between the agar pad and the bottom of the Petri dish (**b**). **b** Petri dish in which the fungal inoculum and the bioreporter agar pad were placed at a distance of ≈1 mm. The microscopy observation area (0.4 × 0.8 mm) for pH mapping during fungal colonisation was at 2.5 mm distance from the fungal inoculum. **c** Fluorescence micrograph of the observation area and corresponding pH heatmaps generated by spatial data interpolation with R programming.

The miniaturised laboratory microcosm allowing for real-time, spatially-resolved detection of in vivo fluorescence signals from individual *Synechocystis* sp. PCC6803_peripHlu cells is described in Table. 1. The setup consisted of a bioreporter agarose pad and a fungal inoculum pad that were placed in close proximity on a glass-cover bottom dish, allowing for ratiometric microscopic observation of the pH-sensitive emission signals (I_510–395_ and I_510–475_) of the individual bioreporter cells upon hyphal colonisation. The circular agarose bioreporter pad (Ø: 18 mm; h: 1.5 mm, Fig. 1a) was prepared as described by Young et al. [42]. using the YBG 11 medium (Supplementary Table S2) with pH = 6.9 (measured with an electrode, SevenExcellence, Mettler-Toledo, Shah Alam, Malaysia) and 1.5 % low-melt agarose (Carl Roth, Karlsruhe, Germany). Briefly, 0.4 mL of warm agarose medium was placed on a circular glass support (Ø: 18 mm, ibidi, Gräfelfing, Germany), immediately covered by another circular glass cover slide (Fig. 1a, Ø: 18 mm), and allowed to cool for 10 min. It then was flipped over, and the circular glass support was removed using tweezers. Five µL of the bioreporter suspension (OD_750_ = 0.45) were evenly pipetted on the agarose pad surface, allowing for a cell coverage of the surface of ≈ 2200 cells mm^2^. After 10 min, the bioreporter pad was flipped over and attached to a glass-cover bottom Petri dish (µ-Dish 35 mm, low, ibidi, Germany) so that the bioreporters were sandwiched between the bottom of the Petri dish and the glass cover shielded agarose pad (Fig. 1b). A circular agarose pad with the fungal inoculum was then placed at 1 mm distance from the bioreporter pad. To prevent possible position shifts of the bioreporter pad during time-lapse monitoring, the whole Petri dish was filled with luke-warm, liquefied YBG 11 low-melt agarose medium (1.5 % agarose, pH 6.9), and the agarose was allowed to cool down and solidify for 1 h. Ratiometric fluorescence signals (I_510–475_ and I_510–395_) of single bioreporter cells were then monitored microscopically for 48 h at constant temperature (25 °C) as described below. Setups in the absence of a fungal inoculum were used as controls. To chemically quantify the pH changes in the presence of hyphae and confirm the functioning of the cell-based bioreporter, a commercially available pH-sensitive optometric sensor foil (SF-HP5R, PreSens, Regensburg, Germany) for non-invasive mapping of pH via a VisiSens TD Detector Unit DU02 (PreSens, Germany) was used (cf. SI and Supplementary Fig. S1 for the calibration procedure of the sensor foils and calibration data). Briefly, a pH sensor foil (1 × 1 cm) was glued to the aforementioned glass-cover bottom Petri dish using silicon glue (PreSens, Germany). The Petri dish was kept in the dark to let the silicon glue cure overnight. A circular agarose pad without bioreporters (Fig. 1a) was placed on top of the sensor foil, and the sensor foil was sandwiched between the bottom of the Petri dish and the glass cover that shielded the agarose pad for 3 h to allow the sensor foil to equilibrate. After that, an agarose pad (Ø 8 mm) with the fungal inoculum was placed at 1 mm distance from the sensor foil. The whole Petri dish was filled with luke-warm low-melt agarose and allowed to cool down for 1 h. Thereafter, images of an observation area (1.8 × 2.5 mm, at a distance of 2.5 mm from the fungal inoculum) were taken by the detector unit (exposure 1,000,000 µm, gain 17) with a 1 h imaging interval during the 48-hour incubation.

### Time-lapse fluorescence microscopic imaging

**Fig. 2:**
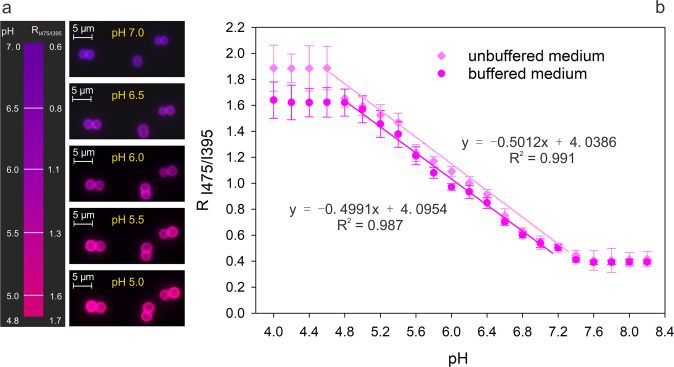
Calibration of the pH bioreporter at the single-cell level. **a** Overlay of fluorescence micrographs of the 510 nm emission signals of *Synechocystis* sp. PCC6803 bioreporter cells after excitation at 475 nm (I_510-475_) and 395 nm (I_510-395_). **b** Calibration curve of emission intensity ratio (R_I475/I395_) and pH obtained in unbuffered and buffered YBG 11 agar pads adjusted to pH 4.0–8.2. Data represent average and standard deviation of >300 cells at each pH.

Ratiometric fluorescence signals (I_510–475_ and I_510–395_) of individual bioreporter cells were monitored using an automated inverted Zeiss microscope equipped with a Colibri LED fluorescence excitation unit (Axio Observer, Carl Zeiss Microscopy GmbH, Jena, Germany). A customised fluorescence filter set was used for fluorescence imaging (AHF Analysentechnik AG, Tübingen, Germany). An area of ~0.4 × 0.8 mm at a distance of 1.5 mm from the edge of the bioreporter pad (i.e. at 2.5 mm from the edge of the fungal inoculum) (Fig. 1b) was used to monitor fungal colonisation and corresponding changes in the 510 nm emission intensity ratio at 395 nm and 475 nm excitation in individual *Synechocystis* sp. PCC6803_peripHlu bioreporter cells. In time-lapse experiments, bright-field (with LED illumination, exposure time 100 ms, light source intensity 4.7 Volt) and fluorescence images at 510 nm emission wavelength after 475-nm (exposure time 100 ms, light source intensity 0.5 Volt) and 395-nm excitation (exposure time 200 ms, light source intensity 0.5 Volt) were taken at 1 h intervals. The focus was kept constant during the time-lapse experiments with an IR-based autofocus system (Definite.Focus 2, Carl Zeiss Microscopy GmbH, Germany). In fluorescence micrographs, red and blue were used as pseudo-colours to represent fluorescence emission excited at 475 nm and 395 nm, respectively. The micrographs show the overlay of the two emission signals leading to pH-dependent changes of the bioreporter pseudo-colours, i.e. purple cells at pH 7, pink at pH 6, and red at pH 5 as indicated by the colour bar (Fig. 2a). All microscopic images were taken at a total magnification of 1000×, employing an oil-immersion objective lens Plan-Apochromat 100x/1.40 Oil Ph3 M27 (Carl Zeiss Microscopy GmbH, Germany). Aforementioned exposure time and LED light intensities as the adjusted parameters for minimising photo-toxicity and fluorescence bleaching during time-lapse microscopy were kept constant for all experiments.

### Image analysis and spatial data interpretation

The I_510–475_ and I_510–395_ of individual bioreporter cells were analysed with ImageJ (https://imagej.net), following a common protocol for cell fluorescence intensity analysis (cf. https://imagej.net/Image_Intensity_Processing as detailed in the SI). After that, the R_I475/I395_ of the individual bioreporter cells were calculated and transformed to pH values by using an independent pH calibration curve obtained from the environmental pH sensitivity confirmation experiment. The generated time-lapse vector data (>700 cells for 48 h) was then imported and interpolated by spatial data analysis approaches (inverse distance weighted, IDW) using the R software 3.6.0 (cf. Script 1 in the SI for the interpolation). Prior to the interpolation, cross-validation (CV), a commonly used method for accuracy estimation and model selection [43], was performed to evaluate the accuracy of IDW in interpolating the mycosphere pH at different spatial resolutions (1 × 1 µm, 2 × 2 µm, 3 × 3 µm, 4 × 4 µm and 5 × 5 µm). The validation was also performed in R software with R programming (cf. Script 2 in the SI). Validation results (Supplementary Table S4) showed that the IDW method could accurately predict (i.e. CV R^2^ ≥ 0.79; prediction error <4.5 %, Supplementary Table S4) the mycosphere pH at spatial resolutions of ≥3 × 3 µm. Hence, IDW was used for the mycosphere pH interpolation at the finest resolution possible (3 × 3 µm) (cf. Script 1 in the SI).

### Statistical analysis

Statistical analysis was performed using SPSS (version 26). The normality of data was verified with the Shapiro–Wilk’s test. To compare the differences between the two groups, a *t*-test was used. To compare differences between multiple groups, means were compared using one-way ANOVA followed by either the LSD test or Dunnett’s T3 test, depending on whether equal variances were or were not assumed, respectively.

## Results

### Construction and reliability of whole-cell pH bioreporters

We constructed a pH-sensitive, cell-based bioreporter to assess the environmental pH at the microscale. The pH-sensitive variant GFP pHluorin2 [21] was translocated into the periplasm of *Synechocystis* sp. PCC6803 using the Tat pathway-specific targeting sequence [19]. The correct clone of the targeting constructs (pBS-TorpHluorin) was verified by sequencing. Translocation of the pHluorin2 to the periplasm was evidenced by pH-sensitive, ring-like expression of pHluorin2 in the periplasm using wide-field fluorescence microscopy of >300 cells (Fig. 2a; Supplementary Fig. S2) showing pH-dependent fluorescence intensity ratios ranging from R_I475/I395_ = 0.41/0.45 (pH 7.4, buffered/ unbuffered medium) to R_I475/I395_ = 1.57/1.59 (pH 5.0, buffered/unbuffered medium). R_I475/I395_ values further showed linear correlation to the external pH in both buffered (pH 5.0–7.4, R^2^ = 0.987) and unbuffered (pH 4.6–7.6, R^2^ = 0.991) media (Fig. 2b), pointing at a directly proportional response of *Synechocystis* sp. PCC6803 transformants to extracellular pH. Furthermore, a standard deviation of R_I475/I395_ (error bars, Fig. 2b) among >300 single individual cells was minimal in the pH range tested, indicating high reliability even of individual cells to report on local pH: standard deviation of R_I475/I395_ ranged from ±0.01 (i.e. ±0.05 pH units) at pH 7.4 to ±0.10 (or ±0.15 pH units) at pH 5.4 (Fig. 2b). Observed R_I475/I395_ was furthermore found to be independent of the growth phase of the bioreporter culture (Supplementary Fig. S3). Despite high cell densities (≈2200 cells mm^2^), the bioreporter presence did not induce statistically significant pH changes over 48 h in the agarose in controls in the absence of hyphae (Supplementary Fig. S4; Supplementary Video S1). Likewise, highly similar calibration curves in strongly buffered and unbuffered media were obtained (Fig. 2b). The bioreporter cells also remained vital in our microcosm over at least 48 h as evidenced by the increasing fluorescence intensity, and, hence, continuous protein synthesis by the cells (Supplementary Fig. S4).

### Time-lapse pH monitoring during fungal habitat colonisation

**Fig. 3:**
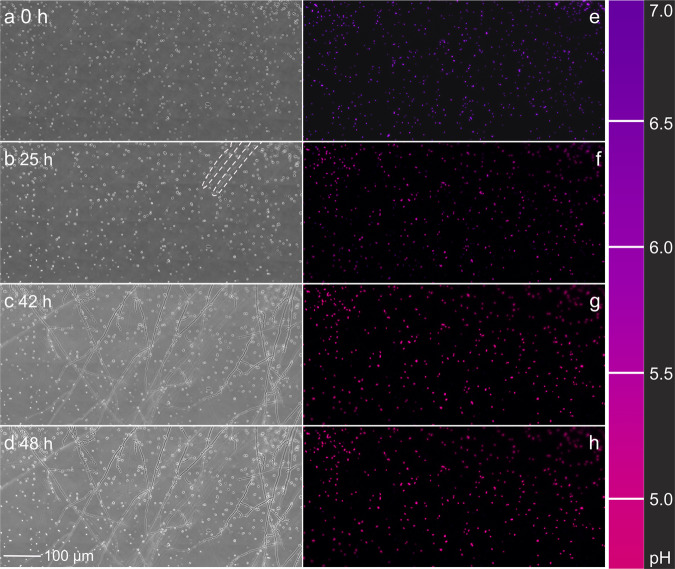
Time-lapse micrographs recording fungal colonisation and bioreporter signals in the observation area. **a**-**d** Bright-field and **e**-**h** fluorescence micrographs of the pH observation area during colonisation of the bioreporter agar pad by *C. cinerea* hyphae over 48 h. Fluorescence micrographs show the overlay of the emission signals of *Synechocystis* sp. PCC6803 cells after excitation at 475 nm (I_510-475_) and 395 nm (I_510–395_), resp. In **b**, for better visibility, the contours of the colonising hyphae are marked by white dash lines.

**Fig. 4:**
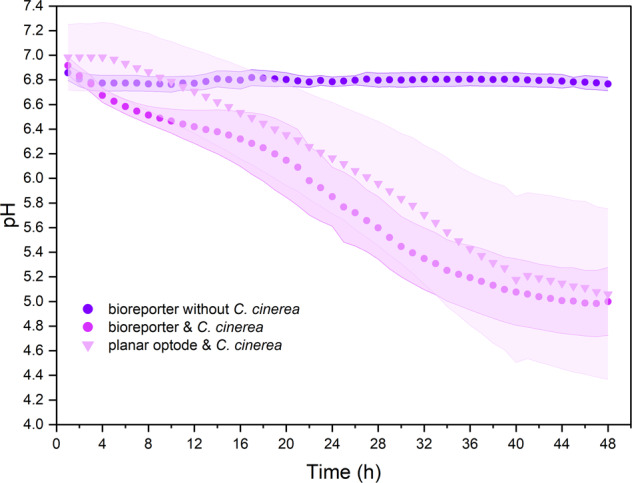
Time dependent development of overall pH in the observation area during the colonisation by hyphae of *C. cinerea*. pH was assessed by *Synechocystis* sp. PCC6803 bioreporter cells and an abiotic planar optode, resp. Bioreporter data encompass the average and standard deviation (shown by shaded error bands) of >700 cells. Experiments in the absence of *C. cinerea* served as controls to analyse the effect of bioreporter cells on environmental pH.

Given the high reliability of individual *Synechocystis* sp. PCC6803 cells to report on extracellular pH, we analysed pH changes during habitat colonisation of the hyphae *C. cinerea* over 48 h by time-lapse microscopic imaging (Fig. 3; Supplementary Video S2). Fig. 3 shows the bright-field images (Fig. 3a–d) and overlay images of 510-nm emission fluorescence signals (Fig. 3e–h) during fungal colonisation. Emission signals changed from purple (Fig. 3e; R_I475/I395_ = 0.58) in the absence of hyphae to magenta (Fig. 3f; R_I475/I395_ = 1.22) in the presence of two hyphal tips after 25 h (Fig. 3b), and finally to red after 42 h (Fig. 3g; R_I475/I395_ = 1.55) in the presence of dense hyphal networks (Fig. 3c). However, after 42 h, no further colour change and fungal colonisation were observed. Based on the R_I475/I395_ of >700 individual cells and standard curves (Fig. 2b, unbuffered media), the average pH in the observation area (0.4 × 0.8 mm) was calculated. Fungal colonisation resulted in a drop of the average pH from 6.9 ± 0.07 to 5.0 ± 0.28 (Fig. 4), while the average pH in mycelia-free controls remained constant (pH 6.8–6.9) over time. Standard deviations (*s* = ±0.07 pH unit, cf. error bars in Fig. 4) among individual cells remained small at any given time in the mycelia-free controls, whereas the pH standard deviations increased from *s* = ±0.10 (*t* = 0 h) to 0.31 at *t* = 21 h (Fig. 4) and 0.28 at *t* = 48 h, indicating high pH variability in the observation area upon fungal colonisation. Biosensor pH data were supported by chemical pH sensing using planar optodes applied to similar microcosms in the absence of bioreporters (Supplementary Video S3). Although optode-derived pH values were 0–0.4 pH units higher than bioreporter pH data (Fig. 4), such differences were not statistically significant (i.e. *P* > 0.05; Fig. 4; Supplementary Fig. S1) and not found at the beginning and the end of our experiment. They hence may rather be the result of varying fungal colonisation of the observation area. Similar endpoints yet slight variations in the pH development were also observed in replicate experiments (Supplementary Fig. S5).

### pH changes in the mycosphere at the microscale

**Fig. 5:**
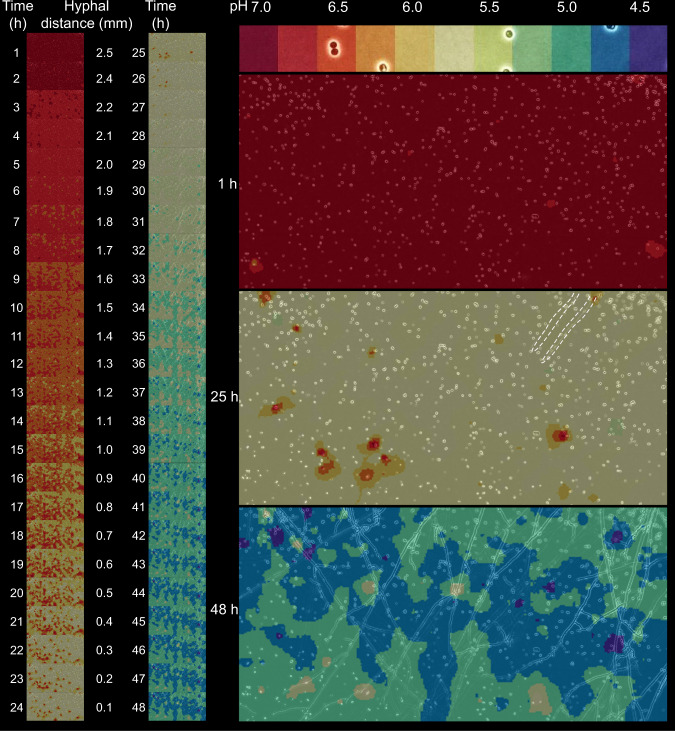
Heatmaps of time-dependent pH changes in the observation area during the colonisation by hyphae of *C. cinerea* over 48 h. The right side of the panel shows heatmaps at *t* = 1, 25 and 48 h. Next to a continuous acidification of the mycosphere, the formation of distinct temporally stable pH gradients is observed. For better visibility, the contours of the colonising hyphae are marked by white dash lines in the *t* = 25 h heatmap.

Time-lapse pH heatmaps (Fig. 5; Supplementary Fig. S6) were created using signals of >700 individual bioreporters in conjunction with IDW interpolation, allowing for a spatial resolution of 3 × 3 µm (cf. materials and methods). Maps of fungus-free control setups showed unchanged and up to the micrometre scale homogeneous pH distribution in the agarose setup over the whole observation period (Supplementary Fig. S6). Observed minor changes (Supplementary Fig. S6) were statistically insignificant (*P* > 0.05). The pH heatmaps (*t* = 1–48 h; Fig. 5) of fungal colonisation of the observation area, however, revealed both a clear pH drop of ~ 2 pH units and increasing heterogeneity of sensed pH by the cells (Supplementary Fig. S7), while pH varied by 0.07 units at *t* = 1–7 h (Supplementary Fig. S7), pH variations later increased to ~0.3 (*t* = 21–48 h, Supplementary Fig. S7) forming temporally stable pH gradients of ≈1.4 pH units over distances of ≈20 µm (Fig. 5, *t* = 42–48 h). As our microcosm allowed for three-dimensional, i.e. spatially intermingled and superimposed growth of hyphae, no direct correlation between pH and hyphal distance was observed in overlays of microscopic images and pH heatmaps (Fig. 5). Our data, however, revealed a pH drop from 6.9 to 6.5 (Fig. 5, *t* = 7 h) before the first hyphae were observed in the observation area (*t* = 25 h, Figs. 3 and 5; Supplementary Video S2).

## Discussion

### *Synechocystis* sp. PCC6803_peripHlu are reliable in vivo pH biosensors for pH mapping

Constructing the novel pH-sensitive bioreporter *Synechocystis* sp. PCC6803_peripHlu, we developed a spatio-temporally resolved approach to quantify the in vivo pH in a microbial habitat at the single-cell scale (~3 µm). Our bioreporter allowed for accurate ratiometric pH detection with a precision of 0.05–0.15 pH units at pH values between 4.6 and 7.4; i.e. a range relevant for the activity of many bacteria and fungi coexisting for example in soil environments where fungi may exhibit wider pH ranges for optimal growth than bacteria [44]. pH detection in our bioreporters is based on the ratiometric pHluorin2, a protein that has been used to sense the intracellular [23–25], though so far not the extracellular (i.e. environmental) pH of a cell. The ratiometric signals of our bioreporter cells responded linearly to environmental pH. They were not influenced by the fluorescence intensity of cells (Supplementary Figs. S4 and S8), the cell growth phase (Supplementary Fig. S3) or intrapopulation heterogeneities (e.g. varying gene expression or reporter protein maturation times [45, 46]). This suggests that results of even individual pH reporter cells can be used to continuously and non-invasively quantify the pH in their immediate microenvironments and, hence, complement chemical or electrochemical pH analyses down to the micrometre (3 × 3 µm) scale. Bacterial life typically thrives in microheterogenic environments [45]. By virtue of the size, single-cell bioreporters have been proven as extremely powerful for analysing microscale environment and micro-gradients (water availability [47], substrate and nutrients [48, 49] and antibiotic gradients [50]). Application of the pH biosensor in microenvironments such as in biofilms, soil microcosms may also possible with the help of advanced microscopy such as confocal laser scanning microscope (CLSM). Such successful application of pH bioreporters enables us to observe pH at a scale relevant for cellular life and, better understand the formation of micro-niche differences (e.g. in biofilms and soils) and their importance to macroscale processes [51, 52].

### pH biosensors allow for time-lapse and microscale mapping of pH during fungal habitat colonisation

The high reliability of individual pH bioreporter cells in conjunction with the possibility to homogeneously immobilise them at high spatial density coverage (≈1.5% or 2200 cells mm^2^) allowed us to continuously map the pH during the colonisation of a habitat by hyphae of the litter decomposing basidiomycete *C. cinerea* [30, 53], a well-characterised model organism [30] with a known genome often used for studying mushroom development and fungal sex and mating types. *C. cinerea* lowered the mycosphere pH to ≈5.0 (Fig. 4; Supplementary Video S3 for planar optode analysis); an acidic pH that has been found to be favourable for the activity of extracellular enzymes of basidiomycetes [54–57]. The pH range and distribution observed in our study (Figs. 4 and 5) also fit well with the exo-enzyme inventory of *C. cinerea*. The genome of *C. cinerea*, encodes for 17 members of the laccase multi-gene family that are likely involved in the degradation of lignin [31, 58] and, the oxidation of phenolic substrates either at acidic (Lcc8 [59], optimal pH of 4.5–5.0) or circum-neutral pH (Lcc1 and Lcc9: [60, 61] pH 6.5), respectively. *C. cinerea* also encodes [31] for lytic polysaccharide monooxygenases (LPMO, demonstrated to cooperate with laccases at optimal pH: 5–5.5 [62]), dye-decolorising peroxidases (DyP; optimal range: pH 3–6), and the *C. cinerea* peroxidase (CiP; optimal range: pH ≈ 7 [63]).

While variations of sensed pH were minor (≤0.1 pH units) in fungus-free controls (Fig. 4 and Supplementary Fig. S6), differences of up to 1.4 pH units were observed during mycelial colonisation (Figs. 4 and 5). This was reflected by spatial pH heterogeneity and the formation of pH gradients of 1.4 pH units over distances of ≈20 µm that remained stable over hours despite high proton diffusion in the aqueous environment (applying Fick’s Law of diffusion and a proton diffusion coefficient of *D*_H+_ = 9.3 × 10^−5^ cm^2^⋅s^−1^, it would take ≈0.08 s for a proton to overcome the gradient). To our knowledge, this is the first experimental report on the formation of temporally stable microscale pH gradients over ~20 µm distances in the mycosphere. We further observed a significant pH decrease from 6.9 to 6.5 at *t* = 7 h and, ≈18 h before the arrival of hyphal tips (*t* = 25 h, Fig. 5; Supplementary Video S2). Our approach allowed us to approximate the width of the zone influenced by hyphal pH change and, using pH as a proxy, to determine zones of hyphal influence in our microcosms. Assuming a hyphal length extension rate of *C. cinerea* of ≈100 µm h^−1^ (cf. Supplementary Fig. S9), a zone of 18 h × 100 µm h^−1^ = 1800 µm width can be approximated. Such range is in line with earlier observations of fungus-induced pH changes at ≈5 mm distance from hyphae at hyphae-soil interfaces [64]. It likewise coincides with the size of oxidation zones around wood colonising hyphae of brown and white rot fungi in the range of several hundred micrometres [65]. Using microfluidic devices [66] allowing for the growth of hyphal monolayers, our bioreporter approach may be used to reveal hyphal foraging strategies by means of pH-dependent exo-enzymes. It may further allow to clarify fungal self-protection mechanisms against reactive oxygen species (e.g. hydroxyl radicals) produced by extracellular Fenton-type reactions around hyphae [32, 67] of wood-rotting basidiomycetes. High concentrations of fungal oxalate (i.e. low pH) in the immediate vicinity of hyphae likely inhibit diphenol-driven Fenton chemistry, while Fenton-type reactions become possible at a higher pH found at larger distances from hyphal surfaces [68, 69]. Micro-scale pH data are hence needed to better understand the functioning of mycosphere habitats and their bacterial-fungal interactions [36, 70, 71]. For instance, complementary roles of deadwood- and straw-degrading white rot and brown rot fungi and N-fixing mycosphere bacteria have been discussed [72]. Bacteria likely depend on mycelial networks [73, 74] and fungal activity for deadwood colonisation and supply of carbon [75]. Finally, as pH may also influence thermodynamics and kinetics of microbial respiration [76] and/or the occurrence and distribution of microorganisms [2–4], bioreporter-based in vivo mapping of microscale pH will enable us to better illuminate the hidden realm of microbial microenvironments.

## Supplementary information

Supplymentry Material

VideoS1

VideoS2

VideoS3

R-Script 1

R-Script 2
