# Supplementary material for: Illuminate the hidden: in vivo mapping of microscale pH in the mycosphere using a novel whole-cell biosensor
Source: ISME Commun. 2021 Dec 11;1:75. doi: 10.1038/s43705-021-00075-3 (PMC9723660; doi:10.1038/s43705-021-00075-3)
Supplement: Supplementary file 1 — Supplymentry Material [file 43705_2021_75_MOESM1_ESM.docx]

**SUPPLEMENTARY INFORMATION**

**Illuminate the hidden: *in vivo* mapping of microscale pH in the mycosphere using a novel whole-cell biosensor**

Bi-Jing Xiong^1^, Christian Dusny^2^, Lin Wang^3,4^, Jens Jappel^5^, Kristin Lindstaedt^2^, Dietmar Schlosser^1^, Hauke Harms^1^, and Lukas Y. Wick^1*^

^1^ Department of Environmental Microbiology, Helmholtz Centre for Environmental Research-UFZ, Permoserstraβe 15, 04318 Leipzig, Germany.

^2^ Department of Solar Materials, Helmholtz Centre for Environmental Research-UFZ, Permoserstraβe 15, 04318 Leipzig, Germany.

^3^ Key Laboratory of Urban Environment and Health, Institute of Urban Environment, Chinese Academy of Sciences, Xiamen, 361021, China
^4^ University of Chinese Academy of Sciences, Beijing, 100049, China

^5^ Department of Biology, Christian-Albrechts-Universität zu Kiel, Am Botanischen Garten 5, 24118 Kiel, Germany.

This supplement contains:

- Information on ‘Materials and Methods’
- 4 Tables
- 9 Figures

Corresponding author: Mailing address: Helmholtz Centre for Environmental Research-UFZ. Department of Environmental Microbiology, Permoserstraße 15, 04318 Leipzig, Germany. phone: +49 341 235 1316, e-mail: [lukas.wick@ufz.de](mailto:lukas.wick@ufz.de).

**Materials and Methods**

**Growth experiment**

*Synechocystis* sp. PCC6803 growth experiments were performed in triplicate in 250-ml flasks. Ten mL pre-culture (OD_750_ = 0.51) were inoculated to 90 mL YBG 11 medium (pH 7.2, buffered with 50 mM HEPES, 100 ppm apramycin) to achieve a starting OD_750_ = 0.05 at t = 0 day. The culture was then grown on a rotary shaker at 150 rpm at 30^o^C and growth followed by OD_750_ measurements. Fluorescent ratiometric response (R_I475/I395_) of the bioreporter cells to pH 7.0 (YBG 11, 50 mM HEPES) were measured on a daily basis (except on weekends) for 16 days (as described in the main text)

**Calibration of optometric sensor foil**

The sensor foil was calibrated in identical setups used for bioreporter-based pH detection: one piece of the chemical sensor foil (1 × 1 cm, SF-HP5R, PreSens, Germany) was glued to the bottom of a glass-cover Petri dish (µ-Dish 35 mm, low, ibidi, Germany) using silicon glue. The Petri dish was then kept in dark to let the silicon glue cure overnight. Thereafter, a standard YBG 11 agar pad (i.e. agar pad with pH = 8.0, unbuffered YBG 11, cf. below) was placed onto the sensor foil and hand-warm, liquefied YBG 11 low-melt agarose medium (1.5% agarose, pH 8.0) was filled to the Petri dish, and the agarose allowed to cool down and solidify for 1 h. Calibration was performed using fluorescence ratiometric imaging (FRIM) with the detector unit (VisiSens TD Detector Unit DU02 (PreSens, Germany) set to an exposure time of 1000000 µs and a gain gate at 17. Similar calibration was performed for each of the pH of 5.0, 5.5, 6.0, 6.5, 7.0, 7.5 and 8.0. Calibration curve (Fig. S1) was built using ratios obtained from the calibration images.

**Preparation of YBG 11 media**

*Preparation of unbuffered medium:* YBG 11 solutions (Table S2, initial pH 7.8) were adjusted to pH values of 4.0, 4.2, 4.4, 4.6, 4.8, 5.0, 5.2, 5.4, 5.6, 5.8, 6.0, 6.2, 6.4, 6.6, 6.8, 7.0, 7.2, 7.4, 7.6, 7.8 8.0, and 8.2 using 2 N H_2_SO_4_ or 2 N NaOH. The pH was measured by a calibrated pH electrode (SevenExcellence, Mettler-Toledo, Shah Alam, Malaysia).

*Preparation of buffered YBG 11 medium:* Citric acid-Na_2_HPO_4_ buffer pairs were used to prepare the buffered standard YBG 11 media of given pH yet identical ionic strength (1). Specifically, YBG 11 solutions (cf. Table S2 for YBG 11 preparation) containing either 0.1 M-citric acid or 0.2 M-Na_2_HPO_4_ were prepared. They then were mixed at ratios as listed in Table S3 to obtain buffered YBG 11 media of given pH between 4.0 and 8.2.

**Analysis of bioreporter cell fluorescence intensity**

Fluorescence images of the 510 nm emission signals of *Synechocystis* sp. PCC6803 bioreporter cells after excitation at 475 nm (I_510-475_; in red pseudo-colour) and 395 nm (I_510-395_; in blue pseudo-colour) were imported by using the image sequence importing function of ImageJ (<https://imagej.net>). The imported colour (RGB) images were first converted to 16-bit greyscale images. The converted image sequence was then duplicated and one copy was used to create binary images by using the threshold function. The created binary images were then used as masks to redirect to analyse cell intensity of the greyscale images (this was achieved by using the ‘Analyze-Set measurements’ command, information on the bioreporters’ cell area, integrated intensity, mean grey value, centroid (cell geographical location) and labels were selected to analyse).

By using the generated binary images as masks, the intensity analysis at single-cell level was finished by running the ‘Analyze-Analyze particles’ command. Information about the bioreporters’ cell area, geographical location and intensity of both I_510-475_ and I_510-395_ were listed on the ImageJ documentation page. I_510-475_ and I_510-395_ were used for R_I475/I395_ calculation, and R_I475/I395_ were transformed to pH value by using the calibration curve in Fig. 1b (unbuffered medium). The pH reported from individual biosensor cells and their centroid information were further used for spatial data interpolation with R programming (c.f. main manuscript, Image analysis and spatial data interpretation).

**10-fold cross validation for accuracy estimation of IDW interpolation and spatial resolution selection**

Cross-validation (CV) is one of the most commonly used methods for data accuracy estimation and model selection (2). 10-fold cross-validation was therefore performed to evaluate the accuracy of the inverse distance weighted (IDW) interpolation method used to interpret mycosphere pH at different spatial resolutions (1 × 1 µm, 2 × 2 µm, 3 × 3 µm, 4 × 4 µm and 5 × 5 µm). The performance of 10-fold CV at each spatial resolution was conducted by the R software 3.6.0 using a script detailed in Script 2. In the validation script, at each spatial resolution, the 48 time-lapse pH datasets were randomly split into 10 subsets D (D1, D2,…, D10) of approximately equal size. The IDW was then trained and tested 10 times; each time, i.e. t = 1, IDW was trained on the nine subsets (D2-D10) and tested on the one left subset (here namely D1). The coefficient of determination (R^2^) and relative prediction error (RPE) of the CV (Table S4) were then calculated according to the overall number of correct/false predictions. Validation results showed that the IDW method could accurately predict the mycosphere pH at all three spatial resolutions above 3 × 3 µm (all R^2^ > 0.75 and RPE < 4.5%, cf. Table S4). Thus the spatial pH data was interpolated with IDW at the finest resolution of 3 × 3 µm.

**Measurement of the hyphal length extension rate**

Time-lapse bright-field micrographs were used for analysing hyphal length extension rate of *C. cinerea*. Microscopic images obtained between t = 25 h (two hyphal tips were first seen in the observation area, Fig. 3b) and 42 h (Fig 3c-3d; Video S3) were used to estimate hyphal length extension rates: visible hyphal extension between every two consecutive time points were checked and marked manually with the freehand function in imageJ (i.e. analysis for time point 30 h and 31 h, Fig. S9), length of all free hand/curved lines were further measured by using “Measure” command in ROI manager in ImageJ. Ultimately, 124 visible extensions (25 - 42 h) were observed and measured, and an average hyphal length extension rate of 101.2 ± 18.6 µm h^-1^ was measured.

**Table S1.** Primers used in this study

| **Primer** | **Sequence** | **Fragment amplified** | **Vector** |
| --- | --- | --- | --- |
| G-5-trc | GGGGATTTATATGGACTCGAaaggcgcactcccgttctgga | *trc*-promoter | pBS-TorpHluorin |
| 3-trc-rbs | CTCGCCCTTGCTCACCATATGATTAATCTCCTtgaaattgttatccgctcaca |  |  |
| N-Luorin2 | AATCATATGGTGAGCAAGGGCGAGGAG | pHluorin2 |  |
| C-luorin2 | tgataaacttatcatccccttttgTCGCCCTTtcaCTTGTACAGC |  |  |
| 5-Apra | CAAAAGGGGATGATAAGTTTATCA | Apramycin resistance |  |
| G-3-Apra | AGTGGATCCCCCGGGCTGCAgagctcagccaatcgactggcga |  |  |
| N-TorA3 | aacaatttcaAGGAGATTAATCATATGAACAATAACGATCT  CTTTCAGG | *torA* sequence with targeting signal |  |
| C-TorA3 | AGCTCCTCGCCCTTGCTCACCACGTCAGTCGCCGCTTGCGCCGCA |  |  |

**Table S2.** Composition of unbuffered YBG 11 medium. 10 mL of the trace elements solution and 10 mL of a 1M glucose solution were added to 980 mL of the minimal YBG 11 medium.

| **Minimal YBG 11 medium** | Concentration (g L^-1^) |
| --- | --- |
| NaNO_3_  MgSO_4_x 7H_2_O  K_2_HPO_4_  Na_2_CO_3_ | 1.49  0.074  0.0305  0.019 |
| **Trace elements solution** | Concentration (mg L^-1^) |
| CaCl_2_ x 2H_2_O  Citric Acid x H_2_O  Boric Acid  MnCl_2_·4 H_2_O  ZnSO_4_.7H_2_O  Na_2_MoO_4_.2H_2_O  CuSO_4_.5H_2_O  Co(NO_3_)2.6H_2_O  FeCl_3_ 6H_2_O  Na_2_EDTA·2H_2_O | 36  6.3  2.78  1.13  0.2  0.39  0.07  0.05  0.97  5.95 |
| **Glucose solution** | Concentration (g L^-1^ = 10 mM) |
| Glucose | 1.80 |

**Table S3:** Citric acid-Na_2_HPO_4_ buffer pairs were used to prepare buffered YBG 11 media. Given pH (4.0 – 8.2) was obtained by adding YBG 11 medium containing 0.1 M citric acid or 0.2 M Na_2_HPO_4_, at ratios as listed below .

| **pH obtained** | **‘X mL’ of 0.1 M citric acid YBG11** | **‘Y mL’**  **of 0.2 M Na_2_HPO_4_ YBG11** |
| --- | --- | --- |
| 4.0 | 61.45 | 38.55 |
| 4.2 | 58.6 | 41.4 |
| 4.4 | 55.9 | 44.1 |
| 4.6 | 53.25 | 46.75 |
| 4.8 | 50.7 | 49.3 |
| 5.0 | 48.5 | 51.5 |
| 5.2 | 46.4 | 53.6 |
| 5.4 | 44.25 | 55.75 |
| 5.6 | 42 | 58 |
| 5.8 | 39.55 | 60.45 |
| 6.0 | 36.85 | 63.15 |
| 6.2 | 33.9 | 66.1 |
| 6.4 | 30.75 | 69.25 |
| 6.6 | 27.25 | 72.75 |
| 6.8 | 22.75 | 77.25 |
| 7.0 | 17.65 | 82.35 |
| 7.2 | 13.05 | 86.95 |
| 7.4 | 9.15 | 90.85 |
| 7.6 | 6.35 | 93.65 |
| 7.8 | 3.75 | 96.25 |
| 8.0 | 1.65 | 98.35 |
| 8.2 | 0.51 | 99.49 |

**Table S4:** The coefficient of determination (R^2^; including intercept and slope) and the relative prediction error (RPE) of the CV 10-fold cross validation used for the accuracy estimation of IDW interpolation and spatial resolution selection.

| Resolution | R^2^ | intercept | slope | RMSE | RPE (%) |
| --- | --- | --- | --- | --- | --- |
| 1 × 1 µm | 0.73 | 0.85 | 0.83 | 0.26 | 5.15 |
| 2 × 2 µm | 0.74 | 0.87 | 0.83 | 0.26 | 5.05 |
| 3 × 3 µm | 0.79 | 0.90 | 0.85 | 0.26 | 4.45 |
| 4 × 4 µm | 0.79 | 0.90 | 0.85 | 0.26 | 4.45 |
| 5 × 5 µm | 0.79 | 0.89 | 0.85 | 0.26 | 4.45 |


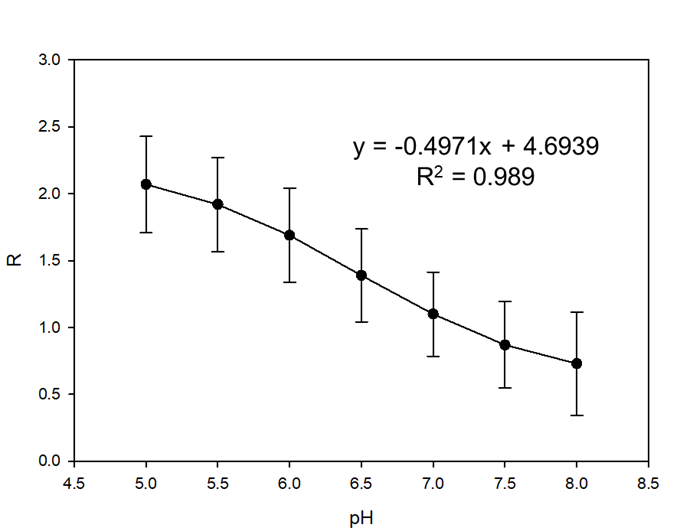


**Figure S1.** Calibration of the chemical optodes (SF-HP5R, PreSens, Germany) at pH 5-8 using fluorescence ratiometric imaging with a commercial detector unit of unknown excitation and emission wavelengths. Data represent average and standard deviation of the optical signals measured in the imaged area (1.8 × 2.5 mm) at each pH level and in triplicate. The standard deviations of the optode ratiometric signal (R) varied from 0.31 to 0.38 at all pH referring to a precision of ± 0.7 and ± 0.8 pH units of the optometric sensor foil.


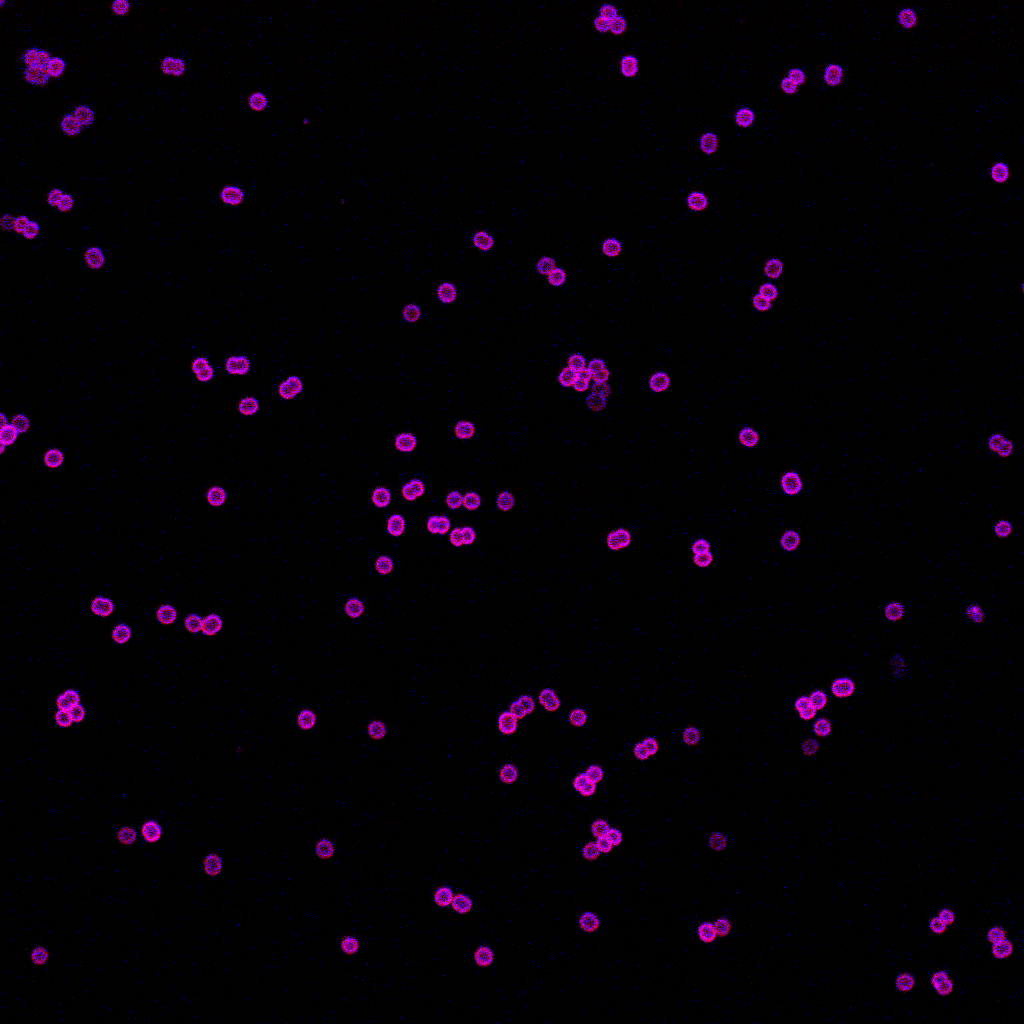


10 µm

**Figure S2.** pHluorin2 protein is almost exclusively located in the periplasm of pH bioreporter cells exposed to medium with a pH of 6.5 (unbuffered YBG 11): the periplasmic rings were observed by fluorescence microscopy. Image shows the overlay of two emission signals excited at 475-nm (exposure time 100 ms, light source intensity 0.5 Volt) and 395-nm excitation (exposure time 200 ms, light source intensity 0.5 Volt) respectively.

**Figure S3.** Growth curve of *Synechocystis* sp. PCC6803 (average and standard deviation of OD_750_ of triplicate cultures; green filled circles) and corresponding R_I475/I395_ at pH 7 (average and standard deviations (0.55 ± 0.02) of > 300 cells for each replicate, pink dots).

**
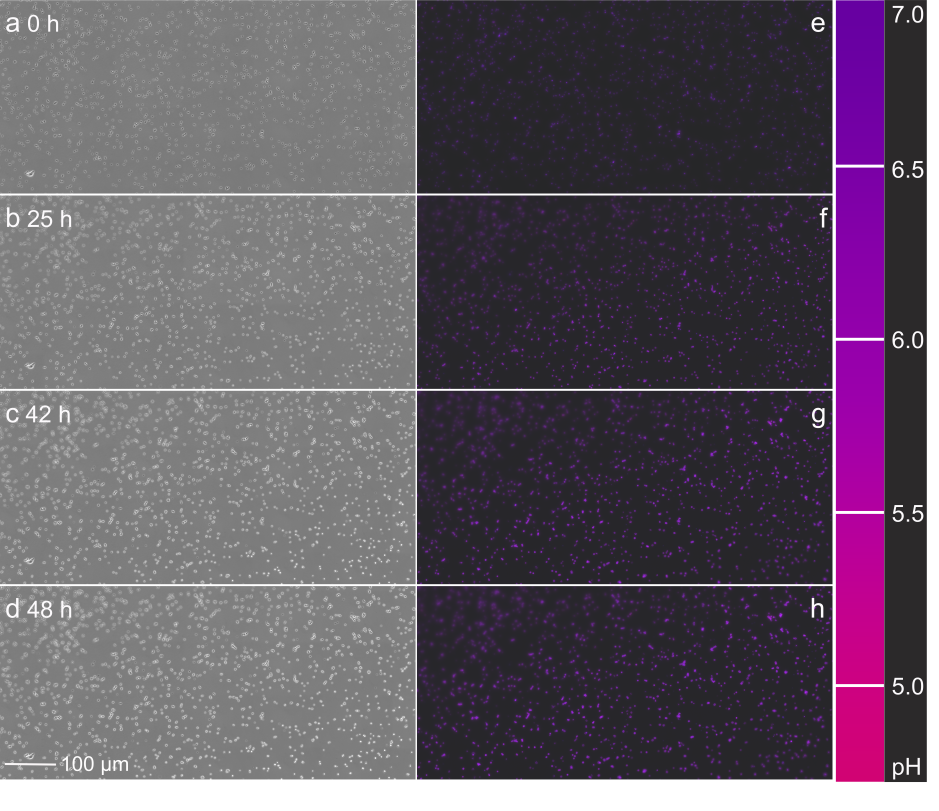
**

**Figure S4.** Bright-field (**Figs. S4a-d**) and fluorescence (**Figs. S4e-h**) micrographs of the pH observation area of a control bioreporter agar pad in the absence of *C. cinerea* observed over 48 h. Fluorescence micrographs show the overlay of the two emission signals of *Synechocystis* sp. PCC6803 cells after excitation at 475 nm (I_510-475_) and 395 nm (I_510-395_). Although overall fluorescence intensity of the bioreporter cells increased during the incubation (cf. increasing cell brightness over time, **Figs. S4e-h**), the R_I475/I395_ remained constant (cf. **Fig. S8**).

**
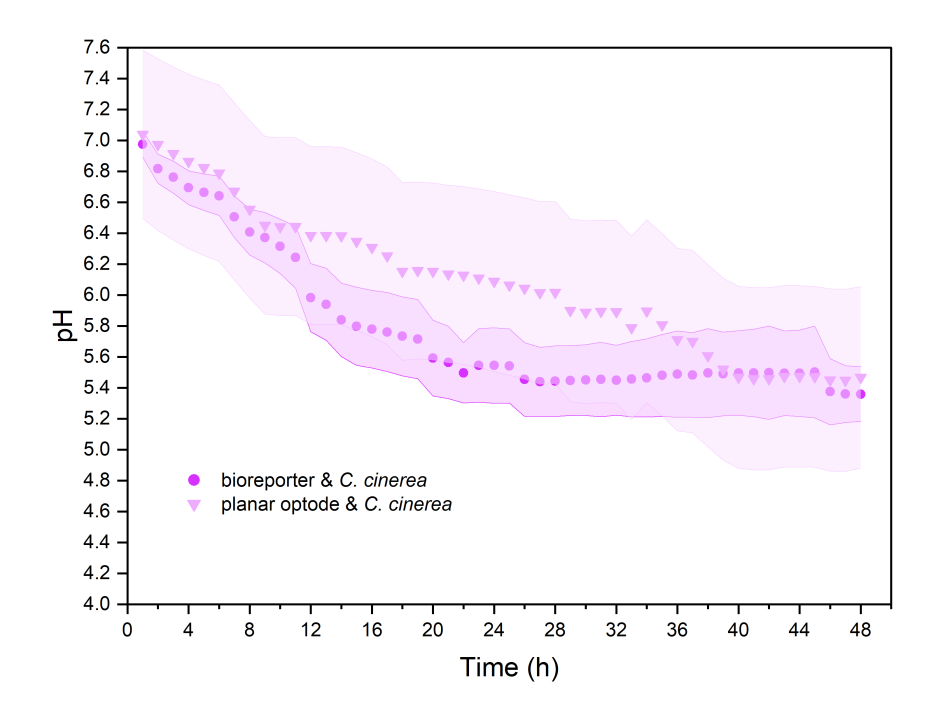
**

**Figure S5**. Time dependent development of overall pH in the observation area during the colonisation by hyphae of *C. cinerea.*  pH was assessed by *Synechocystis* sp. PCC6803 bioreporter cells and an abiotic planar optode, resp. Bioreporter data encompass the average and standard deviation (shown by shaded error bands) of > 700 cells.

**
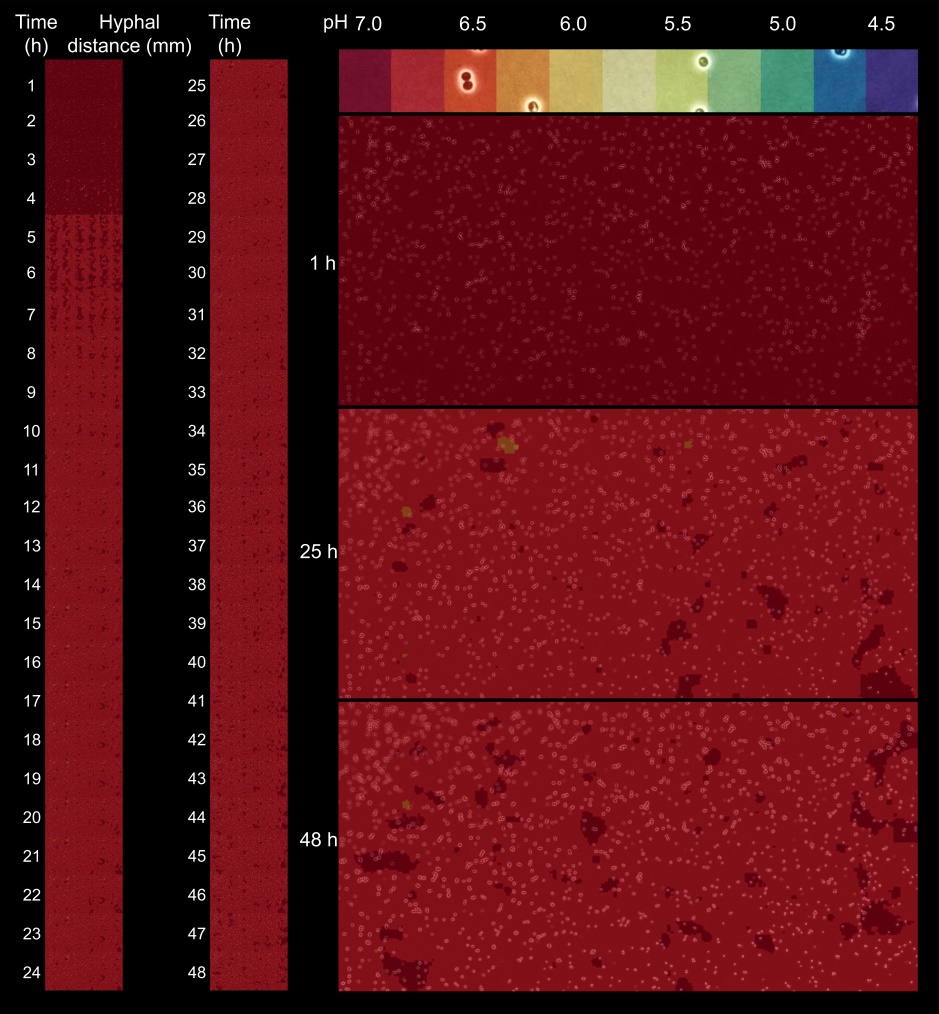
**

**Figure S6.** pH heatmaps of time-dependent pH changes in a control observation area in the absence fungus *C. cinerea* over 48 h. The right side of the panel shows heatmaps at T = 1, 25, and 48 h. Stable pH of 6.8 was observed. Variations of pH (visible by small pH islets of ~7.0 (dark red zones)) were statistically not significant (P > 0.05).

**Figure S7.** Distribution of sensed pH by > 700 *Synechocystis* sp. PCC6803 bioreporter cells in the observation area (0.4 × 0.8 mm) at different times during colonisation by *C. cinerea*.

**Figure S8.** Mean and standard deviation of the fluorescence intensity of > 700 cells at excitation of 475 nm (red filled circles) and 395 nm (blue filled circles) and their ratios (R_475/395_, black diamonds) over time in control experiments in the absence of *C. cinerea*.


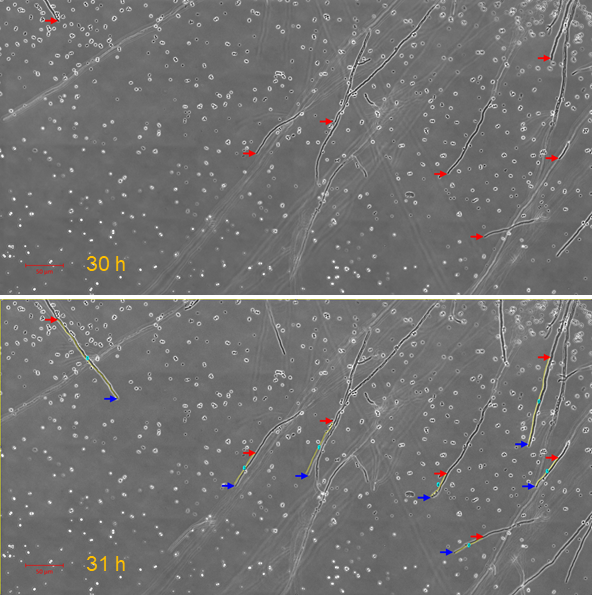


**Figure S9.** An example of hyphal length extension rate measurement (i.e. distance extended between 30 h and 31 h). **a,** red arrows indicate position of hyphal tips at time point 30 h. **b,** blue arrows indicate positions of the extended hyphal tips after 1 h; yellow free hand lines indicate the extended hyphal length. An average extension rate of 101.2 ± 18.6 µm per hour was measured for fungus *C. cinerea* (n = 124 visible extensions in total, 25 - 42 h, cf. Video S3).

**References**

1. Dawson RMC, Elliott DC, Elliott WH, Jones KM. Data for biochemical research: Clarendon Press; 2002.

2. Kohavi R, editor A study of cross-validation and bootstrap for accuracy estimation and model selection. *International Joint Conference on Artificial Intelligence*; 1995: Montreal, Canada.
