# Supplementary material for: Illuminate the hidden: in vivo mapping of microscale pH in the mycosphere using a novel whole-cell biosensor
Source: ISME Commun. 2021 Dec 11;1:75. doi: 10.1038/s43705-021-00075-3 (PMC9723660; doi:10.1038/s43705-021-00075-3)
Supplement: Supplementary file 5 — R-Script 1 [file 43705_2021_75_MOESM5_ESM.docx]

# Script Used for the spatial data interpolation

library(gstat) # Use gstat's idw routine

library(sp) # Used for the spsample function

library(data.table)

library(plyr)

library(rgdal)

library(classInt)

library(RColorBrewer)

library(maptools)

library(mapdata)

library(ggplot2)

library(OIdata)

library(ggsn)

library(sf)

#library(devtools)

#library(REmap)

grid<-data.frame(read.csv('C:\\data\\xiong\\New folder\\ditu.csv',as.is = TRUE))

ExtendedGrid <- grid[,c(2,3)]

coordinates(ExtendedGrid) = ~X + Y

plot<-"C:\\Users\\xiong\\Desktop\\New folder\\"

#i=1

for (i in 1:length(file)){

dir<-paste("C:\\data\\xiong\\xiong1\\")

file<-sort(list.files(dir))

xiong<-data.frame(read.csv(paste(dir,file[i],sep=""),header=T,as.is=T))

names(xiong)[4:5]<-c('X','Y')

coordinates(xiong) = ~X + Y

kc.Ph<-idw(Ph ~ 1,xiong, ExtendedGrid, nmax=4)

idwre<- cbind(grid, kc.Ph$var1.pred)

#plot(grid)

rm(kc.Ph)

colnames(idwre)[4]<-'Ph'

nclr <- 8

plotclr <- brewer.pal(nclr,"Spectral")

plotclr <- plotclr[nclr:1] # reorder colors

class <- classIntervals(idwre$Ph, nclr, style="fixed",fixedBreaks=c(7,6.75,6.5,6.25,6,5.75,5.5,5.25,5,4.75,4.5))

colcode <- findColours(class, plotclr)

png(filename=paste0(plot,"idw",i,".png"))

par(pin=c(5,2.5))

plot (idwre$X, idwre$Y, col = colcode, pch = 15, cex = 0.3, xlab="X", ylab = "Y",main = paste(i))

axis(side=1, at=c(0,100,200,300,400,500,600,700,800),labels=c(0,100,200,300,400,500,600,700,800))

axis(side=2, at=c(0,100,200,300,400),labels=c(0,100,200,300,400))

#axis(1,1:2,xlab)

dev.off()

}
