# Supplementary material for: Illuminate the hidden: in vivo mapping of microscale pH in the mycosphere using a novel whole-cell biosensor
Source: ISME Commun. 2021 Dec 11;1:75. doi: 10.1038/s43705-021-00075-3 (PMC9723660; doi:10.1038/s43705-021-00075-3)
Supplement: Supplementary file 6 — R-Script 2 [file 43705_2021_75_MOESM6_ESM.docx]

#This code is used for idw interpolation and plot for xiongxiong's data

#Lin Wang

library(gstat) # Use gstat's idw routine

library(sp) # Used for the spsample function

library(data.table)

library(plyr)

library(rgdal)

library(classInt)

library(RColorBrewer)

library(maptools)

library(mapdata)

library(ggplot2)

library(OIdata)

library(ggsn)

library(sf)

#library(devtools)

#library(REmap)

grid<-data.frame(read.csv('C:\\Users\\LYNN\\Desktop\\xiong\\Data_Bijing\\Data_Bijing\\grids\\grid 3.csv',as.is = TRUE))

ExtendedGrid <- grid[,c(2,3)]

coordinates(ExtendedGrid) = ~X + Y

dir<-paste("C:\\Users\\LYNN\\Desktop\\xiong\\Raw data\\")

file<-sort(list.files(dir))

i=1

cv1<-c()

for (i in 1:length(file)){

xiong<-data.frame(read.csv(paste(dir,file[i],sep=""),header=T,as.is=T))

names(xiong)[4:5]<-c('X','Y')

#j=1

for(j in 1:nrow(xiong)){

dist <-sqrt((grid$Y-xiong$Y[j])^2+(grid$X-xiong$X[j])^2)

min.idx<-which(dist==min(dist))

xiong$ID[j]<-grid$ID[min.idx]

}

cv<-c()

CV_segments <- cvsegments(nrow(xiong), 10)

#k=1

for(k in 1:10){

xiong.fit1 <- xiong[CV_segments[k]$V,]

Training <- xiong[-(CV_segments[k]$V),][6:7]

coordinates(xiong.fit1) = ~X + Y

kc.Ph<-idw(ph ~ 1,xiong.fit1, ExtendedGrid, nmax=4)

idwre<- cbind(grid, kc.Ph$var1.pred)

#plot(grid)

rm(kc.Ph)

Training1 <- merge(Training, idwre,by ="ID")

names(Training1)[5]<-"predict"

cv<-rbind(cv,Training1)

print(k)

rm(xiong.fit1,Training,idwre,Training1)

}

cv1<-rbind(cv1,cv)

print(i)

}

A<-plot(cv1$ph,cv1$predict)

reg<-lm(cv1$ph~cv1$predict)

summary1<-data.frame(R2=summary(reg)$r.squared, intercept=reg$coefficients[1],slop=reg$coefficients[2])
